# Supplementary material for: Single-dose replicon RNA Sudan virus vaccine uniformly protects female guinea pigs from disease
Source: Nat Commun. 2025 May 6;16:4199. doi: 10.1038/s41467-025-59560-1 (PMC12056027; doi:10.1038/s41467-025-59560-1)
Supplement: Supplementary file 1 — Supplementary Information [file 41467_2025_59560_MOESM1_ESM.pdf]

Supplemental Materials

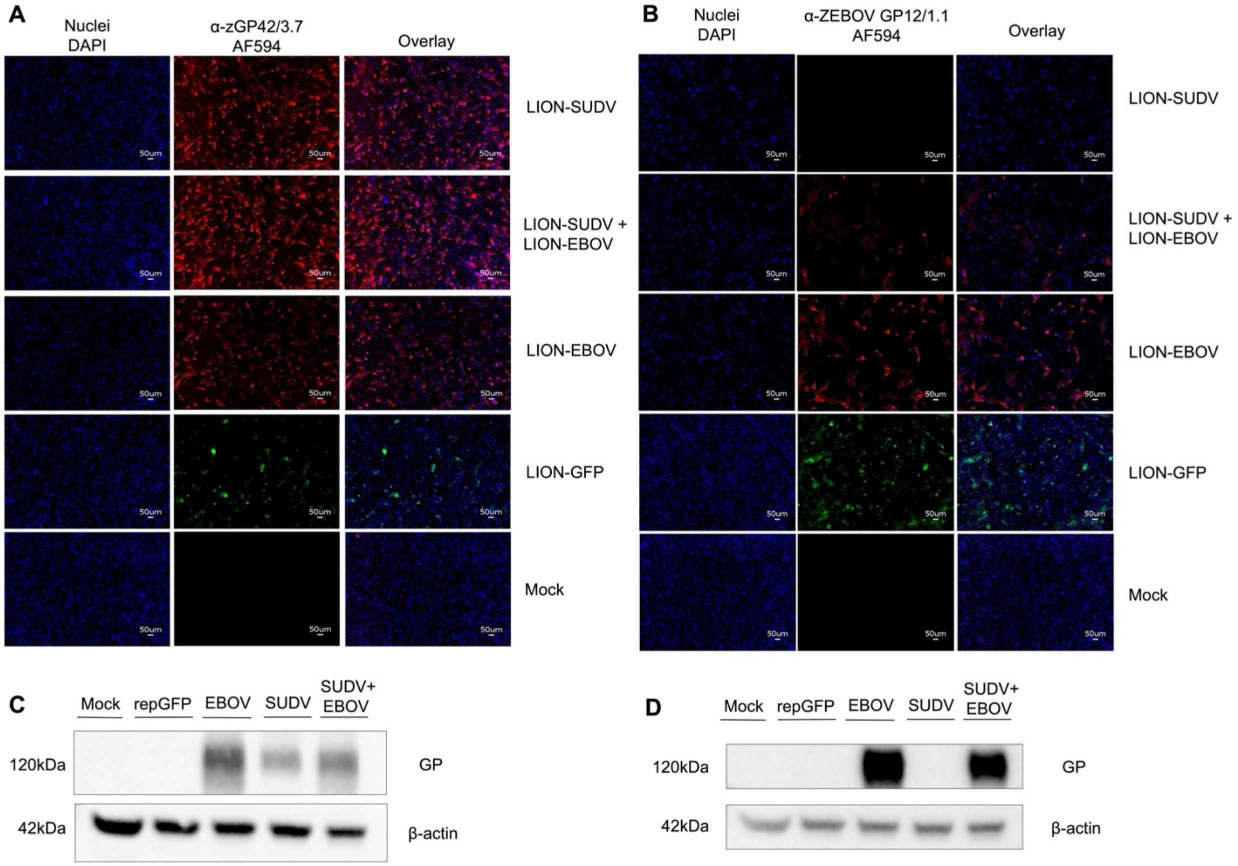

Supplemental Figure 1. Expression of LION vaccine antigens. BHK-21 cells were transfected with the repRNA complexed with LION and harvested for analysis 24 hours later. Filovirus glycoprotein (GP) expression was confirmed by (A, B) immunofluorescence and (C, D) Western blot analysis. The EBOV/SUDV GP cross-reactive antibody ZGP42/3.7 was used in (A, C) and an EBOV GP-specific antibody in (B, D). Experiments were conducted once.

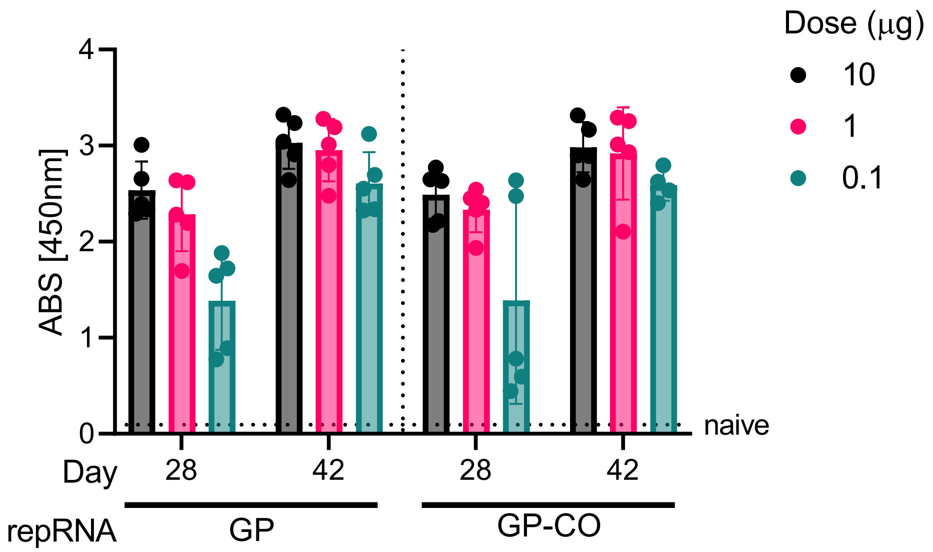

Supplemental Figure 2. Effects of codon-optimization on antigen-specific humoral responses. Wildtype (GP) and codon-optimized (GP-CO) versions of the SUDV GP were used to vaccinate mice (n=5 per group). SUDV GP-specific IgG responses 28- and 42-days post vaccination at three different doses are presented. Data sets display geometric mean and geometric standard deviation and were analyzed using Kruskal-Wallis test with Dunn's multiple comparisons. No significant differences were observed.

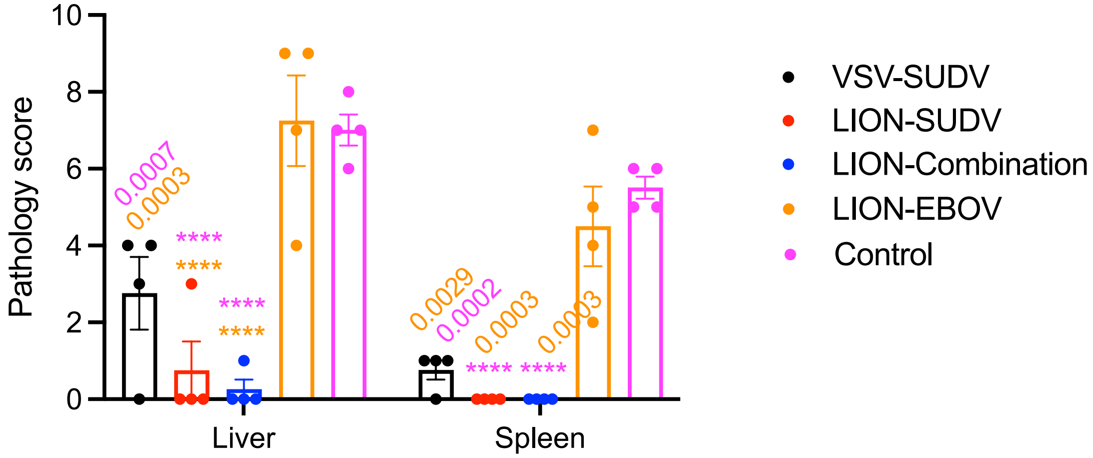

Supplemental Figure 3. Histopathology findings. Histopathologic analysis of liver and spleen samples of vaccinated and control guinea pigs collected 6 days post-challenge (n=4 per group). Depicted are the combined cumulative pathology scores. Data set displays mean and standard error of the mean and was analyzed using two-way ANOVA with. Tukey's multiple comparisons. Statistically significant differences are indicated in colors corresponding to the vaccine group and as **** p <0.0001.

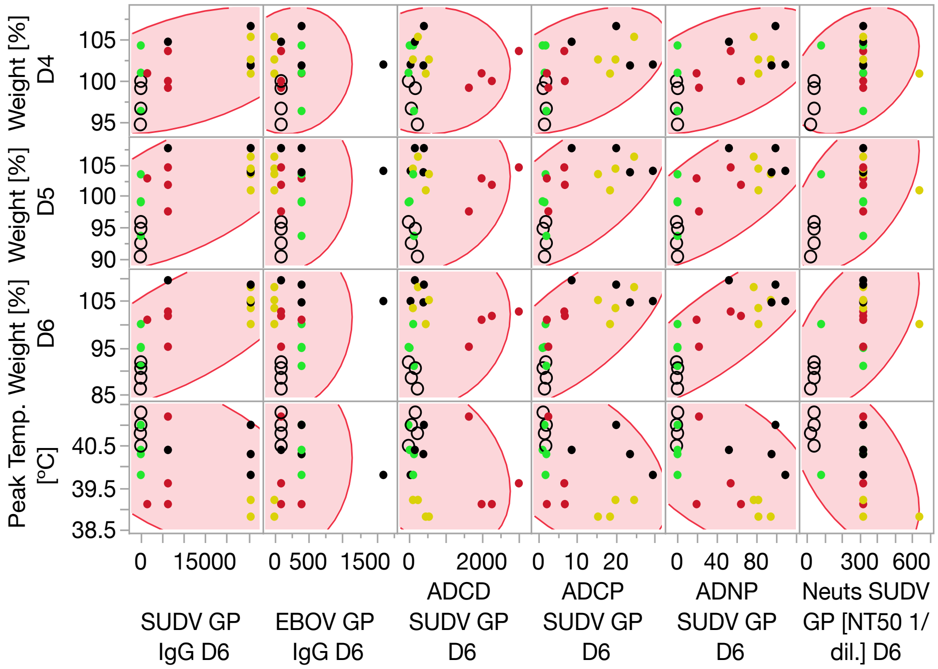

Supplemental Figure 4. Correlation analysis of survival outcomes and antigen-specific humoral responses. Scatterplot matrix showing relationship between clinical symptoms (body weight 4-6 days post-challenge and peak body temperature) and immunogenicity readouts measured on 6 days post-challenge (n=4 per group). Data sets (provided in Supplemental Table s) were analyzed using Spearman's test (two-tailed).

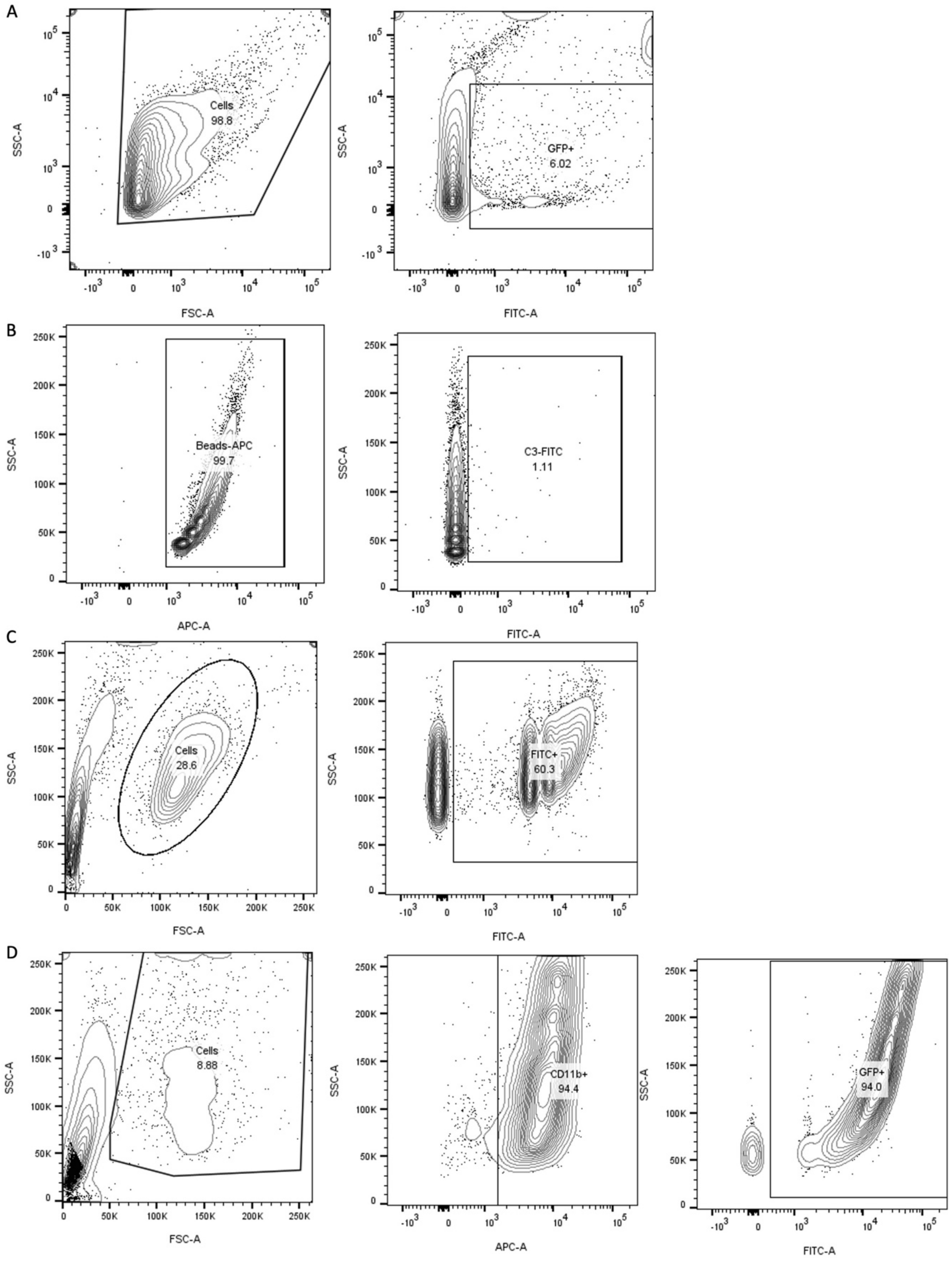

Supplemental Figure 5. Gating strategy for antibody effector function analysis. (A) Neutralization, (B) antibody-dependent complement deposition (ADCD), (C) antibody-dependent cellular phagocytosis (ADCP), and (D) antibody-dependent neutrophil phagocytosis (ADNP).

X	Y	Spearman ρ	p-value
EBOV GP IgG D6	Peak Temp. [ºC]	0.3045	0.1918
ADCD SUDV GP D6		-0.4054	0.0761
ADNP SUDV GP D6		-0.4608	0.0409
ADCP SUDV GP D6		-0.4876	0.0292
SUDV GP IgG D6		-0.5195	0.0189
Neuts SUDV GP [NT50 1/dil.] D6		-0.54	0.014
SUDV GP IgG D6	Weight [%] D4	0.5217	0.0183
ADNP SUDV GP D6		0.471	0.0361
ADCP SUDV GP D6		0.4411	0.0516
Neuts SUDV GP [NT50 1/dil.] D6		0.4028	0.0783
EBOV GP IgG D6		0.0503	0.8331
ADCD SUDV GP D6		0.023	0.9234
SUDV GP IgG D6	Weight [%] D5	0.7581	0.0001
ADCP SUDV GP D6		0.7341	0.0002
ADNP SUDV GP D6		0.7291	0.0003
Neuts SUDV GP [NT50 1/dil.] D6		0.5233	0.0179
ADCD SUDV GP D6		0.2731	0.2439
EBOV GP IgG D6		-0.0395	0.8686
SUDV GP IgG D6	Weight [%] D6	0.839	0.0001
ADCP SUDV GP D6		0.8229	0.0001
ADNP SUDV GP D6		0.8087	0.0001
Neuts SUDV GP [NT50 1/dil.] D6		0.5828	0.007
ADCD SUDV GP D6		0.3341	0.15
EBOV GP IgG D6		-0.0811	0.7341

Supplemental Table 1. Spearman analysis of clinical outcomes and antigen-specific humoral responses. Spearman correlation coefficients (ρ) and two-tailed p-values highlighting relationships that are statistically significant ($p < 0.05$). No adjustments were made to the data prior to analysis.
