# Supplementary material for: Single-dose replicon RNA Sudan virus vaccine uniformly protects female guinea pigs from disease
Source: Nat Commun. 2025 May 6;16:4199. doi: 10.1038/s41467-025-59560-1 (PMC12056027; doi:10.1038/s41467-025-59560-1)
Supplement: Supplementary file 2 — Reporting Summary [file 41467_2025_59560_MOESM2_ESM.pdf]

nature portfolio

Corresponding author(s): Andrea Marzi

Last updated by author(s): Jan 10, 2025

Reporting Summary

Nature Portfolio wishes to improve the reproducibility of the work that we publish. This form provides structure for consistency and transparency in reporting. For further information on Nature Portfolio policies, see our Editorial Policies and the Editorial Policy Checklist.

Statistics

For all statistical analyses, confirm that the following items are present in the figure legend, table legend, main text, or Methods section.

n/a	Confirmed	
☐	☒	The exact sample size (*n*) for each experimental group/condition, given as a discrete number and unit of measurement
☐	☒	A statement on whether measurements were taken from distinct samples or whether the same sample was measured repeatedly
☐	☒	The statistical test(s) used AND whether they are one- or two-sided<br>*Only common tests should be described solely by name; describe more complex techniques in the Methods section.*
☒	☐	A description of all covariates tested
☐	☒	A description of any assumptions or corrections, such as tests of normality and adjustment for multiple comparisons
☐	☒	A full description of the statistical parameters including central tendency (e.g. means) or other basic estimates (e.g. regression coefficient) AND variation (e.g. standard deviation) or associated estimates of uncertainty (e.g. confidence intervals)
☐	☒	For null hypothesis testing, the test statistic (e.g. *F*, *t*, *r*) with confidence intervals, effect sizes, degrees of freedom and *P* value noted<br>*Give P values as exact values whenever suitable.*
☒	☐	For Bayesian analysis, information on the choice of priors and Markov chain Monte Carlo settings
☒	☐	For hierarchical and complex designs, identification of the appropriate level for tests and full reporting of outcomes
☒	☐	Estimates of effect sizes (e.g. Cohen's *d*, Pearson's *r*), indicating how they were calculated

Our web collection on statistics for biologists contains articles on many of the points above.

Software and code

Policy information about availability of computer code

Data collection	N/A
Data analysis	N/A

For manuscripts utilizing custom algorithms or software that are central to the research but not yet described in published literature, software must be made available to editors and reviewers. We strongly encourage code deposition in a community repository (e.g. GitHub). See the Nature Portfolio guidelines for submitting code & software for further information.

Data

Policy information about availability of data

All manuscripts must include a data availability statement. This statement should provide the following information, where applicable:

- Accession codes, unique identifiers, or web links for publicly available datasets
- A description of any restrictions on data availability
- For clinical datasets or third party data, please ensure that the statement adheres to our policy

All data generated and analyzed during this study are included in the manucsript and its supplementary information files.

Research involving human participants, their data, or biological material

Policy information about studies with human participants or human data. See also policy information about sex, gender (identity/presentation), and sexual orientation and race, ethnicity and racism.

Reporting on sex and gender	N/A
Reporting on race, ethnicity, or other socially relevant groupings	N/A
Population characteristics	N/A
Recruitment	N/A
Ethics oversight	N/A

Note that full information on the approval of the study protocol must also be provided in the manuscript.

Field-specific reporting

Please select the one below that is the best fit for your research. If you are not sure, read the appropriate sections before making your selection.

[x] Life sciences   [ ] Behavioural & social sciences   [ ] Ecological, evolutionary & environmental sciences

For a reference copy of the document with all sections, see nature.com/documents/nr-reporting-summary-flat.pdf

Life sciences study design

All studies must disclose on these points even when the disclosure is negative.

Sample size	Five mice were needed to assess differences in absorbance values of humoral immune responses assessed by power analysis using a two-sample t-test with an alpha value of 0.05 and a power of 0.8.<br>Power analysis with the assumption that 100% (99.9999%) of the animals in the control group and 25% of the animals in a vaccine group succumb to challenge would require group sizes of 6 to achieve a significant difference with 0.8 power and an alpha value of 0.05 using Fisher's exact test one-sided hypothesis.<br>Four guinea pigs per necropsy group were required to assess differences in immune responses and virus loads appropriately, reaffirmed by one-way variance power analysis with an alpha value of 0.05 with a power of 0.8.
Data exclusions	No data was excluded.
Replication	The experiments were performed once.
Randomization	Animals were randomized in excel prior to vaccination and assigned numbers for grouping and downstream sample and data processing.
Blinding	All experiments were performed without blinding. Experiments were conducted by the same people with blinding the study being not an option.

Reporting for specific materials, systems and methods

We require information from authors about some types of materials, experimental systems and methods used in many studies. Here, indicate whether each material, system or method listed is relevant to your study. If you are not sure if a list item applies to your research, read the appropriate section before selecting a response.

Materials & experimental systems

n/a	Involved in the study	
[ ]	[x]	Antibodies
[ ]	[x]	Eukaryotic cell lines
[x]	[ ]	Palaeontology and archaeology
[ ]	[x]	Animals and other organisms
[x]	[ ]	Clinical data
[x]	[ ]	Dual use research of concern
[x]	[ ]	Plants

Methods

n/a	Involved in the study	
[x]	[ ]	ChIP-seq
[ ]	[x]	Flow cytometry
[x]	[ ]	MRI-based neuroimaging

Antibodies

Antibodies used	ELISA:<br>Goat Anti-Guinea pig IgG H&L (HRP) (ab6908 Abcam)<br>Flow Cytometry:<br>FITC-anti complement C3 (antibodies online)<br>APC-anti CD11b (Clone 3G8; BioLegend)
Validation	All antibody clones have been validated by the manufacturer (please refer to the data sheet for details).<br>Antibodies used in flow cytometry experiments were systematically titrated before use to ensure that staining is carried out under saturating conditions.

Eukaryotic cell lines

Policy information about cell lines and Sex and Gender in Research

Cell line source(s)	Vero E6 cells (ATCC, Cat. No. CRL-1586), THP-1 (Male, ATCC, Cat. No. TIB-202), HL-60 cells (Female, ATCC, Cat. No. CCL-240)
Authentication	None of the cell lines used were authenticated further.
Mycoplasma contamination	All cell lines were mycoplasma negative.
Commonly misidentified lines<br>(See ICLAC register)	N/A

Animals and other research organisms

Policy information about studies involving animals; ARRIVE guidelines recommended for reporting animal research, and Sex and Gender in Research

Laboratory animals	Female C57BL/6J mice aged 6-8 weeks (Jackson Laboratories); female Hartley guinea pigs aged 6-8 weeks (Envigo).
Wild animals	N/A
Reporting on sex	The SUDV guinea pig model was developed using female guinea pigs so only female guinea pigs were used.
Field-collected samples	N/A
Ethics oversight	Rocky Mountain Laboratories Animal Care and Use Committee (IACUC), University of Washington Animal Care and Use Committee (IACUC).

Note that full information on the approval of the study protocol must also be provided in the manuscript.

Plants

Seed stocks	N/A
Novel plant genotypes	N/A
Authentication	N/A

Flow Cytometry

Plots

Confirm that:

- [x] The axis labels state the marker and fluorochrome used (e.g. CD4-FITC).
- [x] The axis scales are clearly visible. Include numbers along axes only for bottom left plot of group (a 'group' is an analysis of identical markers).
- [x] All plots are contour plots with outliers or pseudocolor plots.
- [x] A numerical value for number of cells or percentage (with statistics) is provided.

Methodology

Sample preparation	Cell lines remained in single cell suspension throughout.
Instrument	FACS Symphony (BD)
Software	BDFACSDiva<br>FlowJo v10.
Cell population abundance	Assays were conducted with cell lines, HL-60 differentiation was confirmed with CD11b+ expression to 94%.
Gating strategy	Neutralization: Cells>FITC+<br>ADCD: Beads>FITC+<br>ADCP: Cells>FITC+<br>ADNP: Cells>CD11b+>FITC+

- [x] Tick this box to confirm that a figure exemplifying the gating strategy is provided in the Supplementary Information.
